# Evaluation of *piggyBac*‐mediated anti‐CD19 CAR‐T cells after ex vivo expansion with aAPCs or magnetic beads

**DOI:** 10.1111/jcmm.16118

**Published:** 2020-11-22

**Authors:** Li‐Rong Yang, Lin Li, Ming‐Yao Meng, Wen‐Ju Wang, Song‐Lin Yang, Yi‐Yi Zhao, Run‐Qing Wang, Hui Gao, Wei‐Wei Tang, Yang Yang, Li‐Li Yang, Li‐Wei Liao, Zong‐Liu Hou

**Affiliations:** ^1^ Central Laboratory of Yan’an Hospital Affiliated to Kunming Medical University Kunming China; ^2^ Key Laboratory of Tumor Immunological Prevention and Treatment of Yunnan Province Kunming China; ^3^ Kunming Medical University Kunming China; ^4^ Yunnan Cell Biology and Clinical Translation Research Center Kunming China

**Keywords:** artificial antigen‐presenting cells, CAR‐T cells, CD19, *piggyBac*

## Abstract

Adoptive immunotherapy is a new potential method of tumour therapy, among which anti‐CD19 chimeric antigen receptor T‐cell therapy (CAR‐T cell), is a typical treatment agent for haematological malignancies. Previous clinical trials showed that the quality and phenotype of CAR‐T cells expanded ex vivo would seriously affect the tumour treatment efficacy. Although magnetic beads are currently widely used to expand CAR‐T cells, the optimal expansion steps and methods have not been completely established. In this study, the differences between CAR‐T cells expanded with anti‐CD3/CD28 mAb‐coated beads and those expanded with cell‐based aAPCs expressing CD19/CD64/CD86/CD137L/mIL‐15 counter‐receptors were compared. The results showed that the number of CD19‐specific CAR‐T cells with a 4‐1BB and CD28 co‐stimulatory domain was much greater with stimulation by aAPCs than that with beads. In addition, the expression of memory marker CD45RO was higher, whereas expression of exhausted molecules was lower in CAR‐T cells expanded with aAPCs comparing with the beads. Both CAR‐T cells showed significant targeted tumoricidal effects. The CAR‐T cells stimulated with aAPCs secreted apoptosis‐related cytokines. Moreover, they also possessed marked anti‐tumour effect on NAMALWA xenograft mouse model. The present findings provided evidence on the safety and advantage of two expansion methods for CAR‐T cells genetically modified by *piggyBac* transposon system.

## 1 INTRODUCTION

Recently, a considerable amount of research work had grown up around the theme of cancer immunotherapy, which had made its unprecedented progress especially in the field of adoptive cellular transfer.^1^, ^2^ FDA approval had been granted lately for the adoptive transfer of T lymphocytes genetically modified to express a CD19‐directed CAR, hence receiving great attention from patients with B‐cell malignancies.^3^, ^4^

The majority of previous clinical trials related to CAR‐T cell therapy had successfully used lentiviral and retroviral vectors to introduce the CD19‐directed CAR gene into T cells. Although lentiviral and retroviral vectors had high gene transfection efficiency, they suffered persistent safety issues caused by the high copy number of viral genes and huge manufacturing cost. One approach to solve this problem involves the use of non‐viral gene transfer to achieve the enforced expression of the introduced ectopic CAR.^5^ A good example of non‐viral gene transfer systems is the transposons, including the *piggyBac* (PB)*, Sleeping Beauty* (SB11), Mos1 and Tol2 systems.^6^, ^7^, ^8^ In comparison with other transposons, the PB transposon/transposase DNA plasmid system derived from the cabbage looper moth *Trichoplusia ni* was found to have surprisingly higher genomic integration efficiencies in cell lines,^9^ including coordinated cuts and pastes of PB transposon from a plasmid by the PB transposase into TTAA‐tetranucleotide sequences in the cellular genome.^10^ Therefore, PB transposon applied through electroporation could be a simplified yet promising alternative to viral systems in clinical setting as a result of its relative safety.^11^, ^12^, ^13^, ^14^

T cells were activated and expanded by antigen‐presenting cells (APCs), but the number of APCs in patients was far from enough. Therefore, researchers had tried many artificial means to amplify CAR‐T cells in vitro. In many studies, magnetic beads and artificial antigen‐presenting cells (aAPCs) were used to expand CAR‐T cells for human application.^15^, ^16^ aAPCs were divided into cell‐based and non‐cell‐based and could be prepared into target‐selective “off‐the‐shelf” feeder cells according to the antigen targets and co‐stimulatory molecules.^17^, ^18^, ^19^, ^20^ The magnetic beads‐amplified CAR‐T cells would need to be screened with puromycin, but they had no viral genetic risk comparing with lentiviral transfected aAPCs. Each of the two methods used to expand CAR‐T cells in vitro possessed their own advantages. However, as far as we know, there were few studies comparing their abilities to expand CAR‐T cells and form memory cells, as well as the tumoricidal activities.

In the past few decades, anti‐CD19 CAR‐T cells had been developed to the fifth generation, but the majority of clinical trials were still using the second‐generation CAR‐T cells.^21^ In order to establish appropriate aAPCs, we genetically modified K562 cells by transfection with CD19‐GFP along with the co‐stimulatory molecules CD137L, CD86, membrane‐bound IL‐15 and the Fc‐receptor CD64. In the present study, we employed PB system to genetically modify T cells to express CD19‐specific CARs, designed with a 4‐1BB and CD28 co‐stimulatory domain, whose structure belonged to the third‐generation CARs. The CAR‐T cells were propagated by aAPCs and anti‐CD3/CD28 beads, respectively. The expression of CARs, memory cell formation and exhausted molecules PD‐1, CTLA‐4 and LAG‐3 expression in the expanded CAR‐T cells would be detected and quantified. Moreover, their targeted tumoricidal activities were also evaluated in vitro. The molecules involved in apoptotic mechanisms, including IFN‐γ, TNF‐α, IL‐2, perforin, granzyme B, caspase 3 and Bax were analysed. Finally, the in vivo tumoricidal efficacy of CAR‐T was explored in NAMALWA tumour xenografts.

## 2 MATERIALS AND METHODS

### 2.1 CAR construction and generation of CAR encoding

The *piggyBac* transposon/transposase system was used to deliver genes into primary human T cells to trigger the expression of CAR specific to the human CD19 antigen. CAR contained the scFv against human CD19 (clone FMC‐63) and linked to the intracellular domains of CD28, 4‐1BB and CD3ζ through a CD28 transmembrane domain and CD8‐hinge. The CD19‐specific CAR cDNA was synthesized using GenScript. The T_2_A and PuroR sequences were isolated from pCDH‐EF1‐MCS‐T2A‐Puro, and CD19 scFv was inserted into the *piggyBac* transposon vector PB‐EF1‐MCS‐IRES‐Neo to replace MCS‐IRES‐neo section together using Xba1 and Bgl2 restriction sites.

### 2.2 Artificial antigen‐presenting cells

**FIGURE 1:**
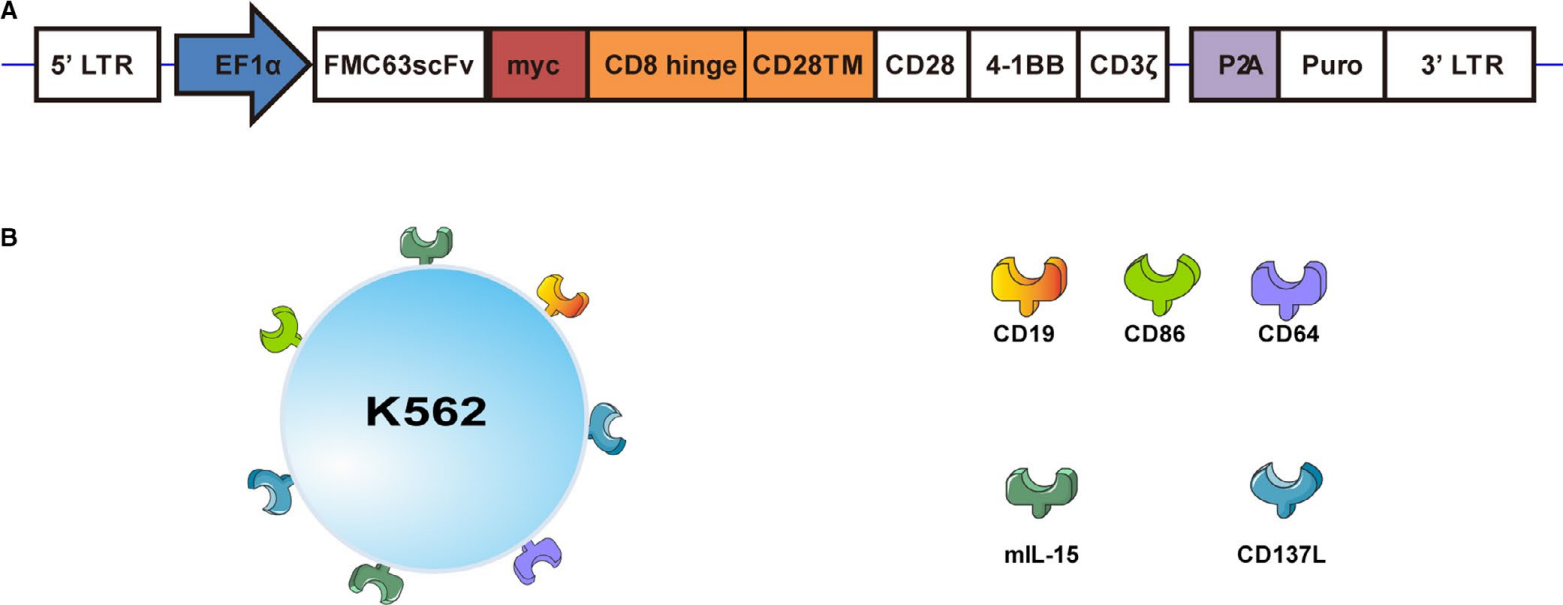
Schematic design of anti‐CD19 CAR‐T cells. A, The third‐generation CAR consisted of the scFv linked to the intracellular domains of CD28, 4‐1BB and CD3ξ through a CD28 transmembrane domain and CD8‐hinge; EF1α, human translation elongation factor‐1 alpha; P_2_A, 2A self‐cleaving peptide derived from the foot‐and‐mouth disease virus (FMDV); Puro, puromycin. B, Artificial antigen‐presenting cells (aAPCs) were derived from K562 cells subjected to transgene transfer via lentiviral vector and clonal selection. Each aAPC clone expressed CD19 (co‐expressed with GFP), CD86, CD64, membrane‐bound interleukin (IL)‐15 and CD137 ligand (CD137L)

K562 cells were purchased from Kunming Institute of Zoology and were cultured in RPMI1640 medium (Gibco, China) supplemented with 10% v/v foetal bovine serum (FBS), 100 U/ml penicillin and 10 U/ml streptomycin at 37°C in a 5% CO_2_, 95% humidified atmosphere. K562 cells were transduced with lentivirus to co‐express CD19 (co‐expressed with GFP), CD86, CD64, membrane‐bound interleukin (IL)‐15 and CD137 ligand (CD137L). They had undergone a series of procedures including viral transfections, monoclonal, amplification and gamma irradiation (Figure 1B), after which they were used as aAPCs for ex vivo expansion of genetically modified T cells.

### 2.3 Electroporation and propagation of CAR‐T cell

Fresh human buffy coat from healthy donors was obtained from Kunming Blood Center, Yunnan Province. The use of human buffy coat for the experiment was approved by the ethics committee of Yan'an Hospital Affiliated to Kunming Medical University (2017‐074‐01). Peripheral blood mononuclear cells (PBMCs) were isolated using Ficoll‐Paque medium (Amersham Biosciences, USA) according to its instruction. On Day 0, PBMCs were suspended in 100 µL of human T‐cell Nucleofector solution (Lonza, Germany) and mixed with 10 µg of transposon PB‐19 CAR‐puro and 5 µg of transposase, or 10µg empty plasmid PB‐T_2_A‐puro and 5 µg of transposase as a control (NT). Then, the samples were electroporated (2 × 10^7^ cells/cuvette) using Nucleofector II—Program U‐14 (Lonza, Germany). After overnight incubation in X‐vivo 15 medium (Lonza, Belgium) at 37℃, the cells were activated with Dynabeads Human T‐Activator CD3/CD28 (Thermo Fisher, Lithuania) (beads: cells = 2:1) or γ‐irradiated (100 Gy) aAPCs ( aAPCs: cells = 2:1) in X‐vivo 15 medium with 300 U/ml IL‐2 (ProteinTech, USA) and 30 ng/ml IL‐21 (ProteinTech, USA) at a density of 10^6^ cells per ml. Stimulation with beads or aAPCs was performed twice during the expansion of CAR‐T cells on days 2 and 12 (or 10). Each stimulation of beads was kept for 72 h, and then, the beads were removed.

The medium was changed every other day. On days 14‐16 or 18‐21, T cells were counted and analysed by FACS for expressions of CAR, memory phenotypic characterization and exhaustion markers.

### 2.4 Detection of the residual status of aAPC after ex vivo expansion

To evaluate the safety of CAR‐T cells propagated with aAPCs, the residual status of aAPCs in the culture system was detected by flow cytometry and PCR. aAPCs was transfected with CD19 lentivirus containing the GFP gene. The expression of GFP can be detected by flow cytometry. The expression of LTR sequence of the lentivirus from aAPCs cells was also tested by PCR. DNA was extracted from cryopreserved aAPCs, CAR‐T cells co‐cultured with aAPCs, PBMC and NT co‐cultured with aAPCs by Wizard Genomic DNA Purification Kit (Promega, USA). To quantify the expression of the LTR sequence of lentivirus, PCR was carried out with C1000 Touch Thermal Cycler (Bio‐Rad, USA). The sequences of primers were described in Table S1. The PCR products were separated by 1% agarose gel electrophoresis and observed by Bio‐Rad GelDoc XR (Bio‐RAD, USA).

### 2.5 Cell line culture

Human burkitt's lymphoma cell lines Raji and NAMALWA were obtained from ATCC, USA, and maintained in RPMI 1640 medium supplemented with 10% or 20% FBS, 100 U/ml penicillin and 10 U/ml streptomycin at 37°C in a 5% CO_2_, 95% humidified atmosphere. Luciferase‐expressing cell lines were generated through inducing lentiviral supernatant into the parental cell line according to reference.^22^

### 2.6 Flow cytometry

In order to analyse the expression of CAR and memory phenotype in CAR‐T cells, cultured CAR‐T cells were collected on the 14‐16 or 18‐21 days for further assessment by flow cytometry. In addition, the molecular expressions of CD86, CD64, CD137L and mIL‐15 on the genetically modified aAPCs were also verified by flow cytometry. The CAR‐T cells or aAPCs (1 × 10^6^ cells) were incubated with the antibodies in flow buffer supplemented with 1% BSA for 15 min at 4°C after washing. Then, the cells were resuspended in flow buffer and analysed by flow cytometry (Cytomics FC 500, Beckman Coulter, USA). The antibodies against CD86‐, CD64‐, CD137L‐, CD45RA‐, CD45RO‐, CD62L‐, PD‐1‐PE, CD44‐, CD3‐, CD4‐, CD8‐PerCP‐Cy5.5, CD56‐FITC and CD19‐BB700 were purchased from BD (Becton Dickinson and Company, USA). The mAb mIL‐15‐PE was bought from R&D systems, USA. The expression of CD19‐specific CAR was detected using Myc‐PE antibody (Cell Signaling Technology, USA). Kaluza Analysis software version 2.1 was used to analyse the data obtained.

### 2.7 Cytotoxicity assays

The cytotoxicity of CAR‐T cells was determined by the cell‐based bioluminescence assay. In brief, 5 × 10^3^ NAMALWA, Raji, K562 and K562 ^CD19+^ cells expressing firefly Luciferase (fLuc+) were cultured in X‐vivo 15 medium in the presence of different ratios of transduced T cells in a 96‐well microplate (BD Biosciences, USA). After incubation for 24 h at 37°C, each well was filled with 100 μL luciferase substrate (Promega, USA) and was measured with a microplate spectrophotometer (Varioskan Lux, Thermo Scientific, USA). Target cells alone were plated at the same cell density to determine the maximal luciferase expression (in relative light units, RLUmax). The following equation was used to calculate the percentage cell viability: [% cell viability = (RLUsample ‐ RLUblank)/ (RLUmax ‐ RLUblank) × 100%]. All data were expressed as mean values of triplicate wells. The whole assay was carried out independently for three times using three batches of CAR‐T cells.

### 2.8 qRT‐PCR analysis

Total RNA was isolated using the typical TRIzol reagent extraction method. cDNA was synthesized using the RevertAid First Strand cDNA Synthesis Kit (Thermo Scientific, USA) according to the manufacturer's instructions. qPCRs were performed with SsoFast^TM^ EvaGreen Supermix (Bio‐Rad, USA) for 35 cycles of 5 seconds denaturation at 95°C followed by 5 seconds annealing/extension at 60°C. Primers for detection of RNA expression were described in Table S1.

### 2.9 Cytokine secretion

CAR‐T cells were added into tumour target cells NAMALWA, Raji, K562 ^CD19+^ and K562 at effector‐to‐target ratios of 2:1 and 4:1 for 24 h. The supernatant was collected and the levels of IFN‐γ, IL‐2, IL‐6, IL‐10, granzyme B, perforin, TNF‐α and GM‐SCF were measured by ELISA according to the kit manufacturer's instruction (NEOBIOSCIENCE, China).

### 2.10 Western blot analysis

All cells were collected and lysed in RIPA lysis buffer (Beyotime, China) supplemented with a protease inhibitor cocktail (Roche Molecular Biochemicals, Switzerland) and phenylmethylsulfonyl fluoride (Beyotime, China). The supernatants were obtained and protein concentration was determined by BCA Protein Assay Kit (Beyotime, China). Proteins (30 or 35 μg) were electrophoresed on a 10%‐12% SDS‐PAGE gel and then electrophoretically transferred to 0.45 μm PVDF membranes (Millipore, Germany). The membranes were blocked with 5% (w/v) non‐fat dry milk in TBS and incubated overnight at 4°C with primary antibodies against Bax, STAT1, caspase 3, cleaved‐caspase 3 and β‐actin (ProteinTech, USA). Then, the membranes were washed with TBS containing 0.1% (v/v) Tween‐20 and incubated with HRP‐conjugated secondary antibodies (ProteinTech, USA). Protein bands were visualized by reaction with chemiluminescent HRP substrate (Millipore, USA) and observed with chemiDoc^TM^ Touch Imaging System (Bio‐Rad, USA).

### 2.11 Scanning electron microscopy

The procedure of seeding and incubating the cells was the same with that for the western blot assay, and all of the cells were suspension‐fixed in glutaraldehyde for 24 h at 4°C and washed with PBS for 3 times. Subsequently, the samples were dehydrated in a graded series of ethanol/water mixture (50% – 100%, v/v) and soaked for 10 min in isoamyl acetate/ethanol mixture (1:1, v/v). After the samples had reached the critical point of drying, they were gold‐sputtered and examined using a scanning electron microscope (Nova NanoSEM 450, FEI, Czech Republic).

### 2.12 In vivo antitumour activity of CAR‐T

Male NOD/SCID mice (6‐8 weeks old) were maintained in a pathogen‐free condition. The animal experiment was approved by the Animal Experimentation Ethics Committee of Yan 'an Hospital Affiliated to Kunming Medical University. The NAMALWA xenograft‐bearing mice model was used to investigate the anti‐tumour efficacies of CAR‐T cells and evaluate the survival rate. Briefly, the mice were first administered with 100 mg/kg cyclophosphamide daily for two days (day −3 and day −2). Then, on day 0, they were infused intravenously with 5 × 10^6^ firefly luciferase‐transduced NAMALWA cells or saline solution (as control). Five days after NAMALWA cells infusion, the tumours were observed through the Xenogen In Vivo Imaging System (IVIS, Caliper Life Sciences, Hopkinton, MA). The mice were randomized into the control group and CAR‐T treatment groups with two different doses. Then, 2 × 10^7^ and 4 × 10^7^ aAPC‐stimulated CAR‐T cells were infused intravenously via tail injection. The tumour bioluminescence was monitored using IVIS on days 5, 12, 19 and 26 (Figure 7A). Furthermore, the survival rate of the mice was recorded during the experiment. According to the requirement of the Animal Ethics Committee, the mice were killed once they developed hind‐limb paralysis or lost 20% of their body weight. Kaplan‐Meier survival curves were generated using the data obtained.

### 2.13 Statistical analysis

All data were compared with unpaired t test or one‐way/two‐way analyses of variance (ANOVA) followed by *post hoc* Tukey's multiple comparison test or Sidak's multiple comparisons test. The overall survival in mice experiment was represented by a Kaplan‐Meier curve, and the survival difference between different groups was compared by a log‐rank test. *P* <0 .05 was considered to be statistically significant. All statistical data were analysed with GraphPad Prism 6 software (GraphPad Software Inc, San Diego, CA).

## 3 RESULTS

### 3.1 Propagation of anti‐CD19 CAR‐T cells after electrotransfer of PB plasmids by co‐culturing with aAPCs or magnetic beads

**FIGURE 2:**
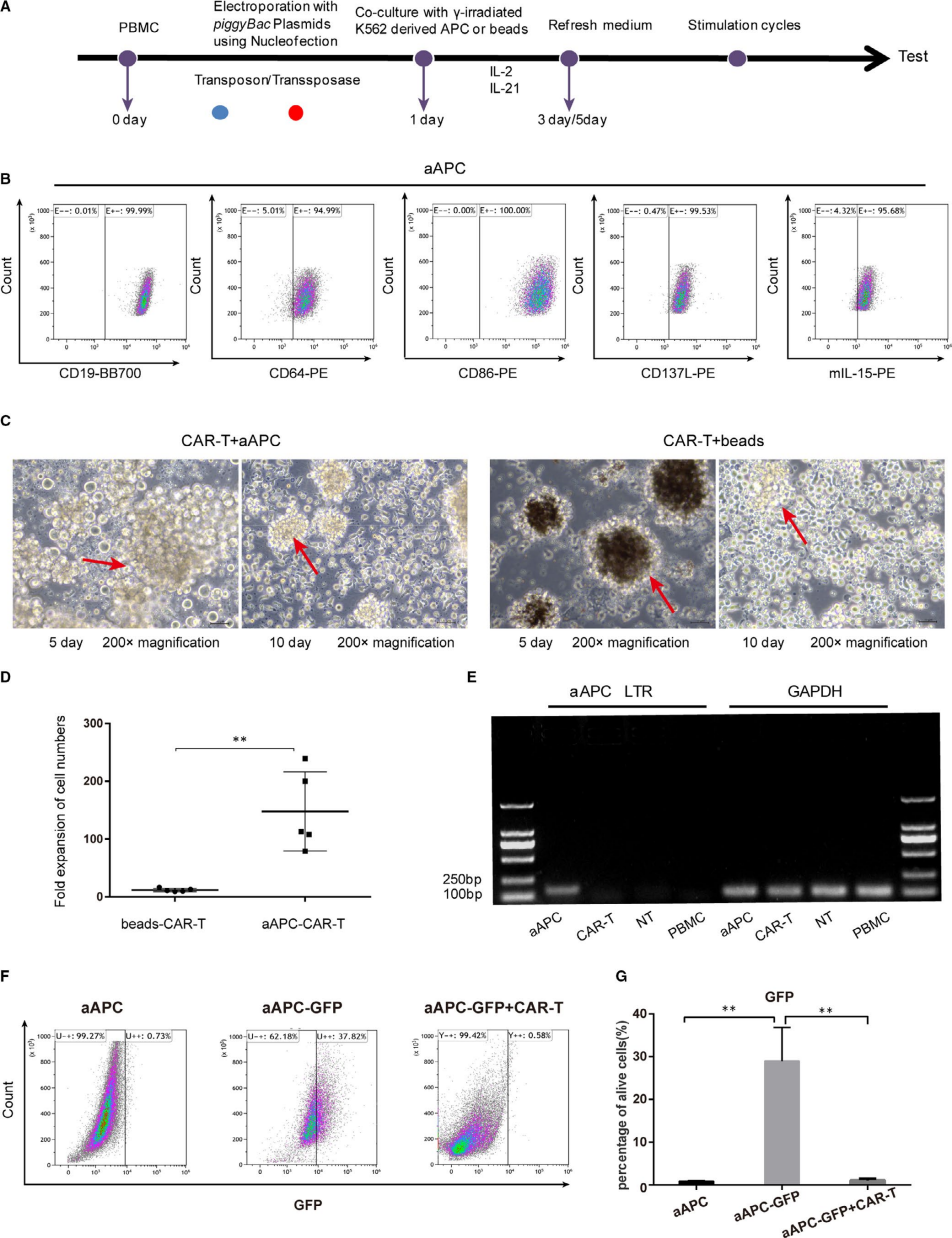
Enrichment and expansion of anti‐CD19 CAR‐T cells with aAPCs or beads. A, On day 0, PBMCs were subjected to synchronous electroporation with DNA plasmid coding for the *piggyBac* transposase plasmid (red) and transposon DNA plasmids coding for CAR species (PB‐19CAR‐puro, blue). To expand T cells stably expressing CARs, genetically modified T cells were co‐cultured with γ‐irradiated aAPCs or magnetic beads in the presence of 300 IU/mL IL‐2 and 30 ng/mL IL‐21 on day 1. Cytokines were added during the replacement of culture medium. Repeated stimulation of expression of CAR with aAPCs or beads occurred every few days. B, The expression levels of CD19 antigen, the co‐stimulatory molecules CD64, CD137L, CD86 and membrane‐bound IL‐15 on cell surface of aAPCs were detected by flow cytometry. C, Representative photographs of CAR‐T cells propagated with aAPCs or beads during various culture days were observed under light microscope. The red arrow indicated T‐cell clones. D, The fold expansion of the number of CAR‐T cells propagated with aAPCs or beads on days 18‐21 or days 14‐16, respectively. The data were calculated as the ratio of the number of cells on the last day to that on the first day. **P < .01, aAPC‐CAR‐T group vs. beads‐CAR‐T group (unpaired t test). The experiment was conducted independently for 5 times. (E) DNA electrophoresis gel diagram. DNA was extracted from aAPCs, CAR‐T cells co‐cultured with aAPCs, PBMCs and NT co‐cultured with aAPCs, and the relative levels of the expression of LTR sequence were assessed by PCR and visualized via gel electrophoresis. (F) GFP expression was monitored by flow cytometry. aAPCs were transfected with GFP, so that the expression of GFP was employed to detect any residue of aAPC after co‐cultured with or without CAR‐T cells. (G) The bar graphs were presented as mean ± SD from three independent experiments. The difference was analysed by unpaired t test. **P < .01 as compared with aAPC‐GFP group

PBMCs were electroporated with DNA plasmids of PB transposon and transposase system to express the third‐generation CAR composed of the FMC63scFv linked to the intracellular domains of CD28, 4‐1BB and CD3ζ through a CD28 transmembrane domain and CD8‐hinge (Figure 1A). aAPCs or magnetic beads were used to stimulate and expand anti‐CD19 CAR‐T cells in the presence of IL‐2 and IL‐21 (Figure 1B, Figure 2A). The results from flow cytometry showed that the expressions of CD19, CD64, CD137L, CD86 and mIL‐15 in the transgenic aAPCs were all more than 90% (Figure 2B).

As shown in Figure 2C, CAR‐T cells were stimulated with aAPCs or magnetic beads, and T‐cell clones were observed under the light microscope (indicated by red arrows). On the 5th day, it was obvious that the CAR‐T cells formed colonies with aAPCs or magnetic beads indicating the cells had good viability and proliferated well over time. On the 10th day, aAPCs were exhausted and CAR‐T cells formed multiple colonies (Figure 2C).

The number of T cells co‐cultured with aAPCs increased by 147.8 ± 30.52 fold after 18‐21 days, whereas that co‐cultured with beads expanded by 11.93 ± 1.324 folds after 14‐16 days (Figure 2D). After the above culture periods, T cells co‐cultured with aAPCs continued to expand, but those with beads stopped proliferating.

To explore the exhaustion of aAPCs in the cultured system, the green fluorescent signal of GFP was successfully detected after transduction of aAPCs by flow cytometry. After co‐cultured with CAR‐T cells for 18 days, GFP expression was nearly undetectable in the cultured system, indicating the aAPCs were severely exhausted (Figure 2F, G). To further evaluate the relative amount of aAPCs, we performed PCR using the DNA of the co‐cultured cells. As aAPCs were modified by lentivirus transfection, we designed a primer to detect the LTR sequence, which might be left as residues of aAPCs. However, gel electrophoresis showed that the PCR product from CAR‐T cells with aAPCs had no LTR sequence band (Figure 2E), indicating there were no aAPCs remaining.

### 3.2 Phenotype of anti‐CD19 CAR‐T cells expanded with aAPCs and beads

**FIGURE 3:**
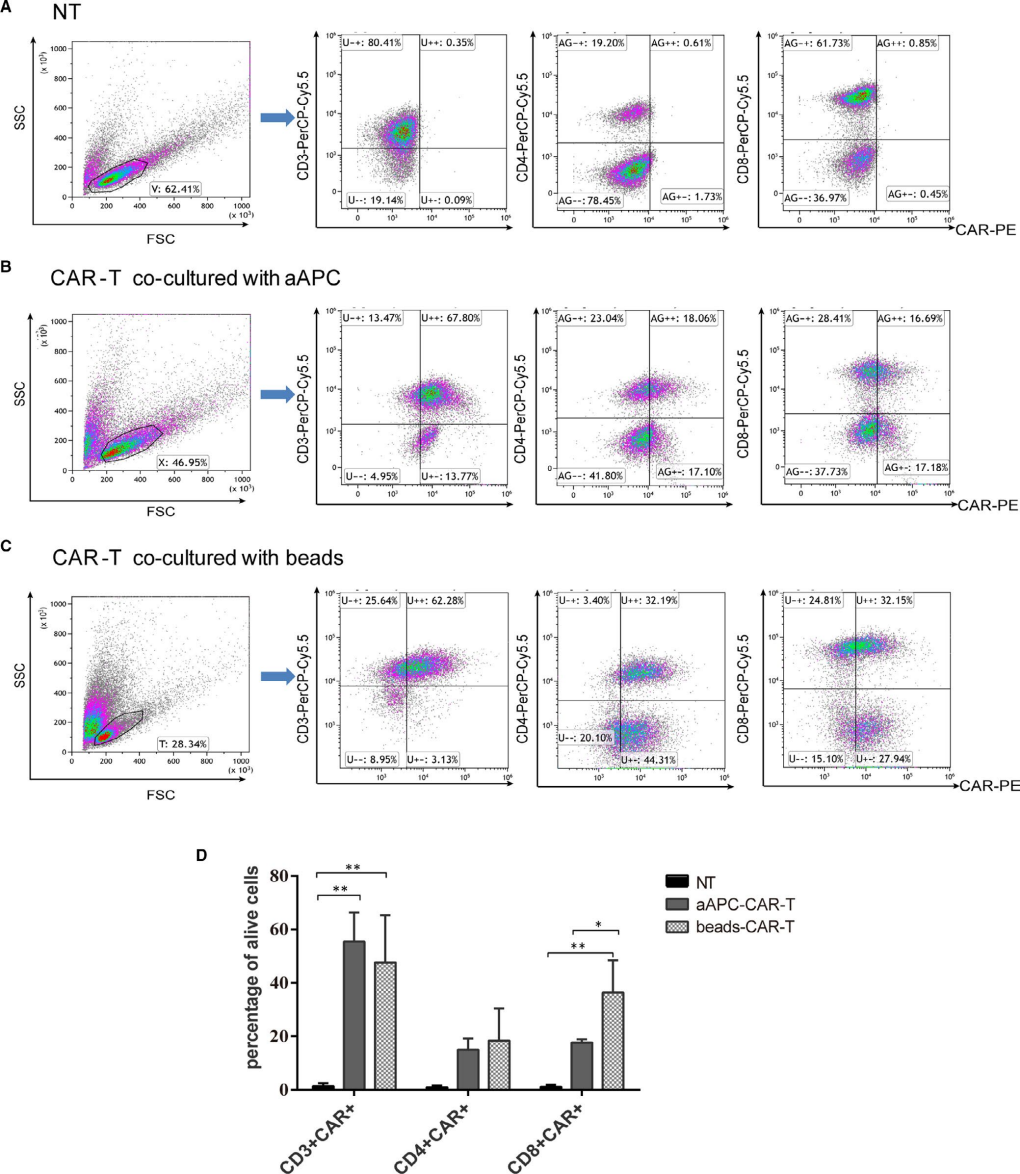
The expression of CD19‐specific CAR on CD3^+^, CD8^+^ and CD4^+^ T cells was assessed by flow cytometry at 18‐21 days or 14‐16 days after propagating CAR‐T cells with aAPCs or magnetic beads, respectively. A‐C, Representative flow cytometry data. D, Bar graphs indicated the mean ± SD from three independent experiments carried out with samples from different blood donors.*P < .05; ***P < .001 as compared with NT group. Differences among all groups were analysed by one‐way ANOVA followed by *post hoc* Tukey's multiple comparison test

To compare the abilities to promote the expression of CAR in alive cells by aAPCs and magnetic beads, the expressions of CD3^+^CAR^+^, CD4^+^CAR^+^ and CD8^+^CAR^+^ were detected by flow cytometry (Figure 3A‐C). The expression of CAR at 24 h after electroporation was 27.28 ± 2.03% (Fig. S1A). Compared with the NT, the aAPCs and magnetic beads groups had significantly higher ratios of alive cells expressing CD3^+^CAR^+^, CD4^+^CAR^+^ and CD8^+^CAR^+^ (Figure 3D). However, there was no difference between the aAPCs and beads groups regarding the rate of alive cells expressing CD3^+^CAR^+^, both of which were above 40%. In addition, we also found that there were some NK cells (CD56^+^CD3^‐^) and NKT cells (CD56^+^CD3^+^) in our cultured cell products, whether with aAPCs or beads stimulation. NK cells accounted for 3.41 ± 2.10% in the alive cells co‐cultured with aAPCs, while only 0.60 ± 0.08% with beads (Fig. S1B&C). However, NKT cells accounted for 8.87 ± 6.18% and 4.74 ± 0.85% in cell population expanded by aAPCs and beads, respectively (Fig. S1B,C). Among them, the positive rate of CAR expression was 66.96%. Therefore, the maximum ratio of CAR‐NKT cells in cell population could reach 14.16% after co‐cultured with aAPCs (Fig. S1B).

The proportion of CD8 ^+^ CAR cells co‐cultured with beads was higher than that with aAPCs. The ratio of CD4^+^/CD8^+^ CAR cells co‐cultured with beads was 0.51, but the ratio of CD4^+^/ CD8^+^ CAR cells stimulated by aAPCs was 0.85 (Figure 3D). The latter resulted in almost equal amounts of CD4^+^CAR^+^ and CD8^+^CAR^+^ T cells, which might be better for clinical treatment.^23^ The results from two‐thirds of trials found that beads preferentially expanded CD8^+^ CAR cells (38.57 ± 11.53) compared with CD4^+^ CAR cells (11.57 ± 2.07).

**FIGURE 4:**
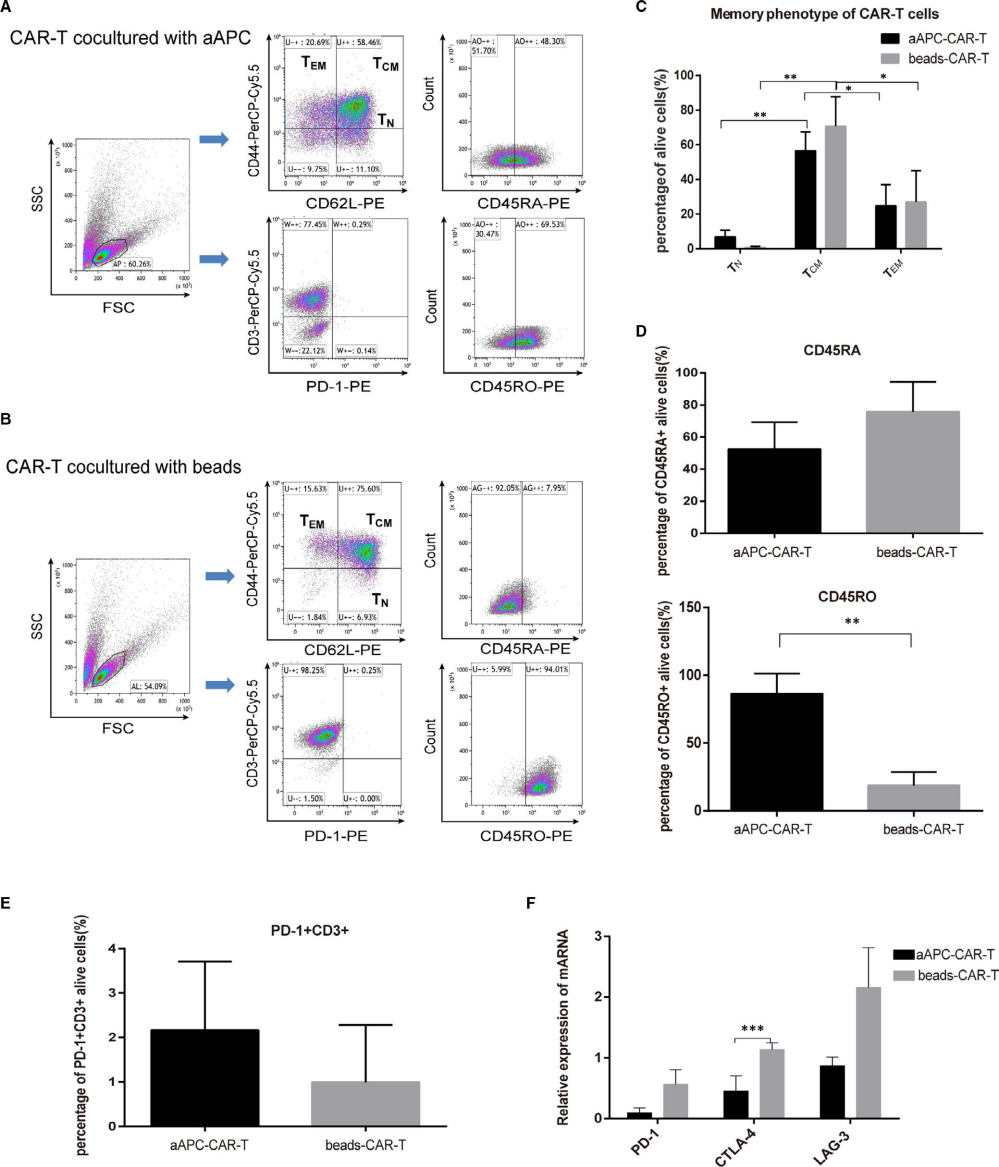
The memory phenotype of CAR‐T cell (CD44, CD62L, CD45RA and CD45RO) and exhaustion phenotype (PD‐1) was determined by flow cytometry at 18‐21 days after propagation of CAR‐T cells with aAPC or at 14‐16 days after propagation of CAR‐T cells with beads. T_N_: CD44^‐^CD62L^+^, T_CM_: CD44^+^CD62L^+^, T_EM_: CD44^+^CD62L^‐^, T_CM_ or T_EM_: CD45RO^+^. (A&B) Representative data from flow cytometry. (C‐E) Bar graphs showed quantified results of flow cytometry. The data were expressed as the mean ± SD from three independent experiments carried out with samples from different blood donors. *P < .05, **P < .01 as compared with T_CM_ or beads‐CAR‐T group by one‐way ANOVA followed by *post hoc* Tukey's multiple comparison test or unpaired t test. (F) The gene expressions of exhausted molecules including PD‐1, CTLA‐4 and LAG‐3 were analysed by real‐time PCR in CAR‐T co‐cultured systems. Data were normalized to corresponding *GAPDH* expressions as internal control. ***P < .001 as compared with beads‐CAR‐T group. Difference between two groups were determined by unpaired *t* test

It is known that adoptive transfer of memory CAR‐T cells would increase long‐lived immune response in vivo.^24^ Therefore, the memory/naïve T cells and expression of exhaustion markers on CD19‐directed CAR‐T cell were compared between the cells co‐cultured with aAPCs and magnetic beads. The percentages of CAR‐T cells expressing effector memory (EM) phenotype (CD44^+^CD62L^‐^) and central memory (CM) phenotype (CD44^+^CD62L^+^) in both ex vivo expansion systems were significantly higher than those of CAR‐T cells which belonged to naïve T cells (T_N_, CD44‐CD62L+)^25^, ^26^ (Figure 4A–C), and the growth of CD45RO^+^ CAR‐T cells (represented T memory cells^27^) stimulated by aAPCs was superior to those expanded with beads. However, there was no difference between the two groups in the expression of CD45RA (Figure 4D). In addition, the percentage of T_CM_ cells from both ex vivo expansion systems was markedly higher than that of T_EM_ (Figure 4D). At the same time, it was found that the expression of PD‐1 was both below 4%. Furthermore, the qPCR results showed that the expression of CTLA‐4 in CAR‐T cells co‐cultured with aAPCs was significantly decreased compared with that co‐cultured with beads (Figure 4E,F). These results indicated that both culture conditions would enhance effector functions of CAR‐T cells, but aAPCs were able to induce stronger memory phenotype and weaker exhaustion expression.

### 3.3 Antitumour efficacy of CD19‐specific CAR‐T cells in vitro

**FIGURE 5:**
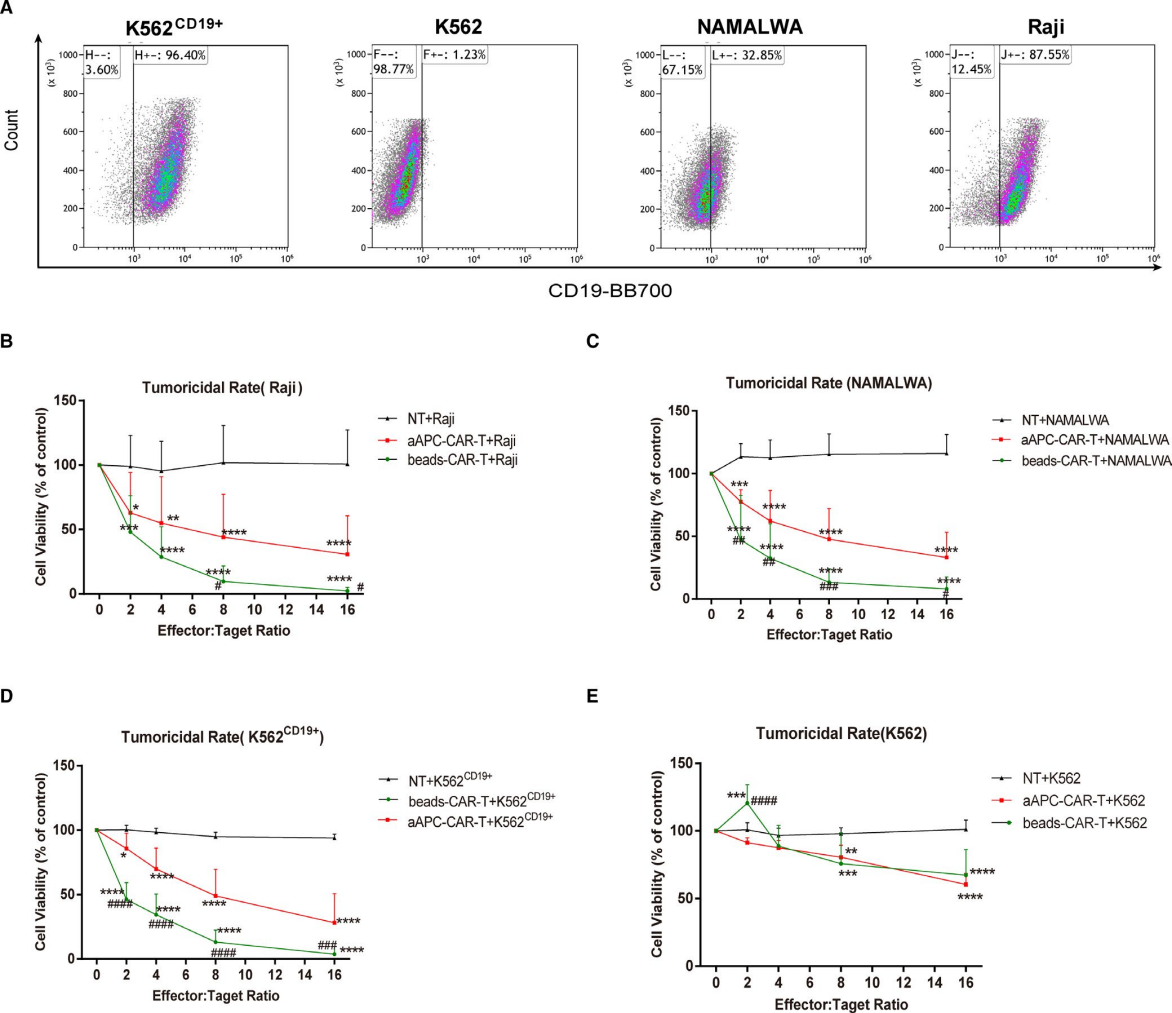
The tumoricidal activities of CAR‐T cells on different haematological cancer cell lines. (A) CD19 expression on NAMALWA, Raji, K562 and K562^CD19+^ cells was detected by flow cytometry. (B‐E) Cytotoxic effects of CAR‐T cells on NAMALWA, Raji, K562 and K562^CD19+^ cells. Cell viability was assessed by cell‐based bioluminescence assay after 24h CAR‐T treatments. Differences between the CAR‐T treated groups and control group (NT) were determined by two‐way ANOVA followed by *post hoc* Tukey's multiple comparison test. **P < .01, ***P < .001 as compared with NT control; ^###^ *P* < .001 as compared with the CAR‐T‐treated bead group. The line graphs represented the mean ± SD from three independent experiments with three wells

As shown in Figure 5A, the intrinsic protein expression of CD19 in NAMALWA and Raji cells was 32.85% and 87.55%, respectively. The expression rate of CD19 in K562 ^CD19+^ cell was 96.40%, which was significantly increased comparing to that in wild‐type K562 (1.23%). CAR‐T cells co‐cultured with aAPCs or beads were able to target CD19^+^ tumour targets specifically in NAMALWA, Raji and K562 ^CD19+^. After 24‐h incubation, CAR‐T cells with both expansion systems significantly induced tumour cell death in all CD19^+^ cancer cell lines in a dose‐dependent manner. However, their tumoricidal effects on K562 cells were lower, indicating the CAR‐T cells were highly specific to CD19^+^ cells. The viability of NAMALWA cells incubated with aAPC‐ and beads‐amplified CAR‐T cells was 47.67 ± 8.125% and 13.31 ± 3.583%, respectively, and that of Raji cells was 44.02 ± 11.08% and 9.64 ± 4.02%, respectively, at an effector‐to‐target ratio of 8:1 (Figure 5B, C). The viability of K562 ^CD19+^ cells was 49.08 ± 6.841% and 13.16 ± 3.095%, respectively, at an effector‐to‐target ratio of 8:1 (Figure 5D). The above results demonstrated that beads‐amplified CAR‐T cells induced higher tumoricidal effects in CD19^+^ cells comparing with aAPC‐amplified CAR‐T cells. Similar results were also observed at effector‐to‐target ratios of 2:1, 4:1 and 16:1. Although CAR‐T cells showed less tumoricidal activities on K562 cells, inhibitory effects were also observed at E:T ratio of 16:1.

### 3.4 Cytokines induced by CAR‐T cells

**FIGURE 6:**
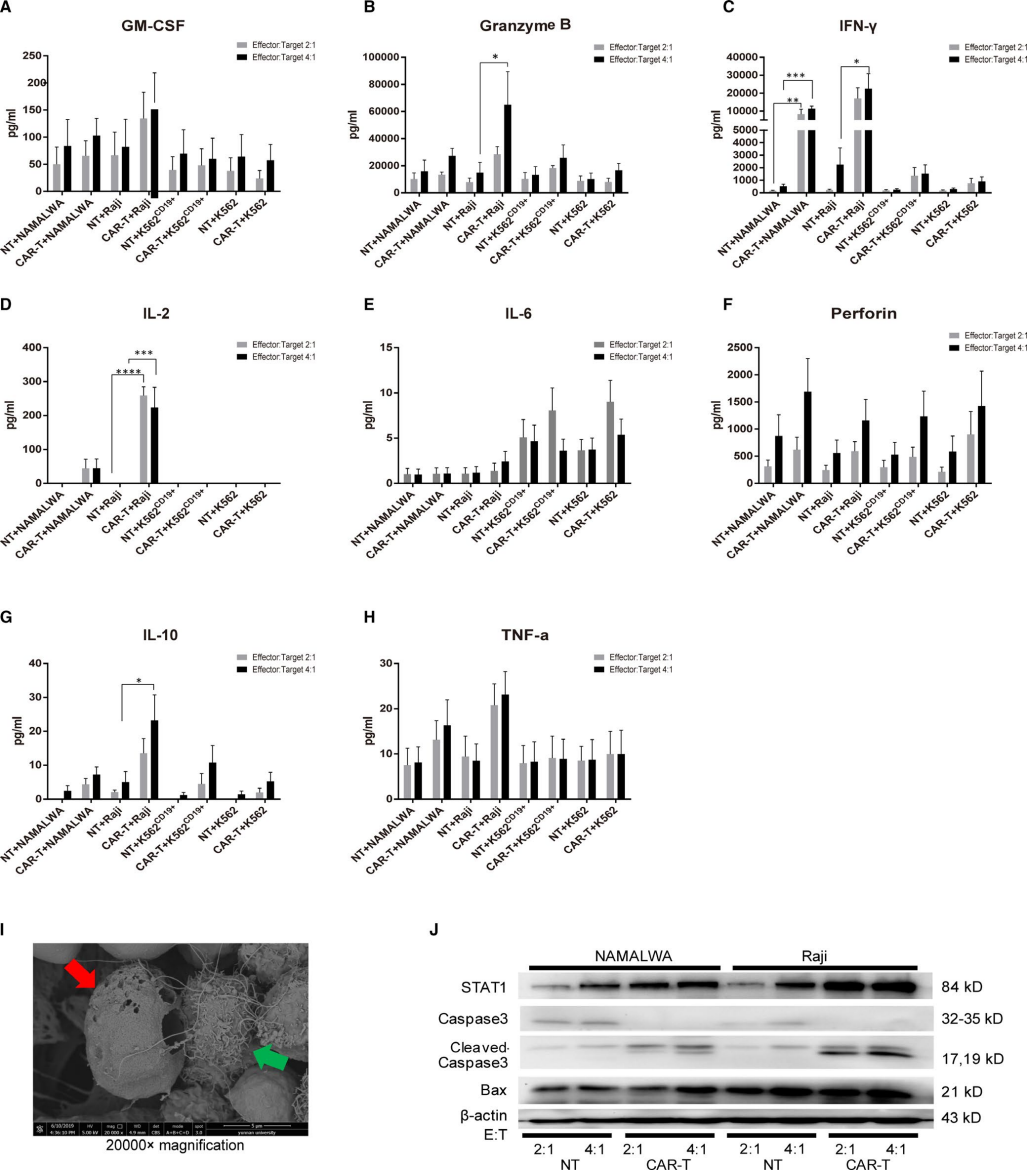
The underlying mechanisms involved in the tumoricidal activities of aAPC‐stimulated CAR‐T cells on different haematological cancer cell lines. (A‐H) The levels of IFN‐γ, TNF‐α, IL‐10, IL‐6, IL‐2, perforin, granzyme B and GM‐CSF in CAR‐T cells co‐cultured with aAPC were detected by ELISA in four different tumour cell lines at different effector‐to‐target ratios.*P < .05,**P < .01, ***P < .001, ****P < .001 as compared with NT control group by two‐way ANOVA followed by Sidak's multiple comparison test. **(**I) Scanning electron micrographs of Raji cells treated with CAR‐T. Green arrow: CAR‐T cell; red arrow: Raji cell. (J) The expression levels of STAT1, caspase 3, cleaved‐caspase 3 and Bax in NAMALWA and Raji cells after treated by aAPC‐CAR‐T cells at different effect‐target ratios were determined by Western blot. β‐actin expression was determined to confirm equal protein loading

To further evaluate the functional properties of CAR‐T cells expanded with aAPCs, levels of a series of cytokines and proteins released from the tumour cells incubated with CAR‐T cells or NT cells were measured. After 24‐h incubation, the levels of IFN‐γ, IL‐2, IL‐10 and granzyme B produced by anti‐CD19 CAR‐T cells were significantly higher than those by NT cells at effector‐to‐target ratios of 2:1 and 4:1 (Figure 6A–H). In addition, the level of perforin was also increased in CAR‐T group comparing with that in NT group. However, there was no significant difference in the release of other immune cytokines including IL‐6, TNF‐α and GM‐CSF. Furthermore, the levels of IL‐10, granzyme B and perforin increased with the increase in the effector‐to‐target ratio.

To further investigate the mechanisms by which CD19**‐**specific CAR‐T cells killed tumours, Western blot analyses of several apoptosis‐related proteins were carried out in lysed NAMALWA and Raji tumour cells incubated with aAPC‐amplified CAR‐T cells. The CAR‐T treatment markedly increased STAT1, cleaved‐caspase 3 and Bax expressions as the effector‐to‐target ratio increased in cancer cells, and the expression of caspase 3 was decreased to undetectable levels after exposure with CAR‐T cells (Figure 6J).

To observe the interaction between CAR‐T cells and tumour cells more intuitively, electron microscopic scan was performed after 24 h co‐culturing of CAR‐T and Raji cells. CAR‐T cells protruded from the pseudopods and enclosed the Raji cells to perforate the cell surface, which showed gradual cracks (Figure 6I).

### 3.5 The antitumour abilities of anti‐CD19 CAR‐T cells in xenograft model

**FIGURE 7:**
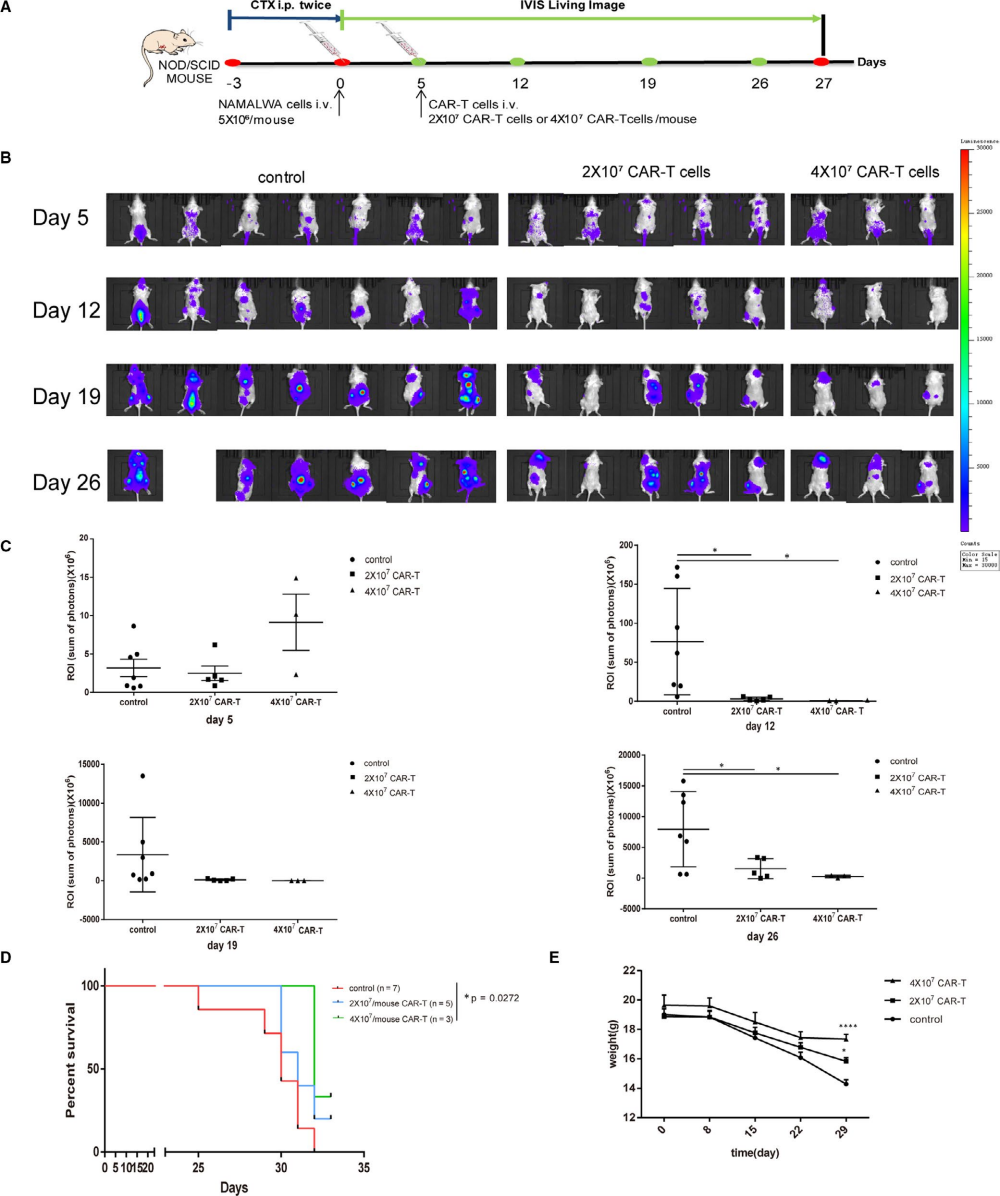
Effects of CAR‐T cells on NAMALWA xenograft mouse model. (A) Schematic diagram of the establishment of the in vivo model. NOD/SCID mice were intravenously injected with NAMALWA tumour cells after intraperitoneal injection of CTX (100mg/kg) for twice. After tumour growth was detected by IVIS, CAR‐T cells were treated intravenously on day 5. The distribution of tumour cells in the mice was monitored weekly by IVIS for three weeks. (B‐D) The anti‐tumour effects of CAR‐T cells on NAMALWA tumour xenografts. (B) Mice were monitored for the distribution of tumour cells with an IVIS Spectrum imaging system after injection of 2 × 10^7^ or 4 × 10^7^ CAR‐T cells (n = 7 for the control group, n = 5 for the 2 × 10^7^ CAR‐T cells group, n = 3 for the 4 × 10^7^ CAR‐T cells group). (C) Quantitative analysis of tumour cells at different time points based on region of interest (ROI) measurements. Data were expressed as mean ± SEM, *P < .05 as compared with control (unpaired t test with Welch's correction). (D) Kaplan‐Meier survival curve of mice bearing NAMALWA tumours (n = 7 for the control group, n = 5 for the 2 × 10^7^ CAR‐T cells group, n = 3 for the 4 × 10^7^ CAR‐T cells group). Data were expressed as the mean ± SEM, *P < .05 as compared with the control (Log‐rank test). (E) The body weight of each mouse was monitored for 29 days. Data were expressed as the mean ± SEM, *P < .05, ****P < .001 as compared with the control by two‐way ANOVA followed by *post hoc* Tukey's multiple comparison test

As anti‐CD19 CAR‐T cells showed promising results in vitro studies, further animal studies were conducted by a xenograft model established by intravenous injection of NAMALWA‐luciferase cells. The NAMALWA tumour xenograft model was successfully established based on IVIS data. The results showed that the tumour burden in mice was significantly reduced in CAR‐T groups comparing with control group (*P* < .01, Figure 7B and C). The tumour burden of certain individuals in the 2 × 10^7^ CAR‐T group had even disappeared completely (Figure 7B). However, there was no significant difference on inhibitory effects between the 2 × 10^7^ CAR‐T group and 4 × 10^7^ CAR‐T group. Moreover, the results showed that survival was prolonged by CD19‐directed CAR‐T cells as compared to control treatment in mice with NAMALWA xenografts. The CAR‐T treatments had a significant delay in disease progression and paralysis, suggesting that CD19 targeting CAR‐T cells could mediate long‐term antitumour responses (Figure 7D). Moreover, there were significant differences in body weight between CAR‐T treatment groups and control at 29th day (Figure 7E). As the disease progress, the body weight of mice was decreased. But CAR‐T treatment also showed some benefits to mice, whose body weight was less reduction comparing with that of control group (Figure 7E).

## 4 DISCUSSION

In the present study, electroporation was employed to prepare CAR‐T cells modified with DNA plasmids of PB transposon and transposase system, in which a high efficiency was observed. The two traditional methods, aAPCs and magnetic beads, to stimulate the expansion of CAR‐T cells had played a significant role in the process. Some molecules were introduced to our aAPCs, which provided diverse stimulated functions. CD137L provides necessary co‐stimulatory signals for activation, proliferation and differentiation of T lymphocytes by cross‐linking with CD137.^17^ CD86 interacts with the corresponding receptor CD28/CTLA‐4 on the surface of T cells to generate co‐stimulatory signals. CD64, a high‐affinity Fc receptor, can also stimulate a variety of immune responses by cross‐linking with IgG Fc, including antigen presentation and the regulation of proliferation and differentiation of T lymphocytes.^28^ IL‐15 promotes the generation of memory T cells and prevents the apoptosis of memory T cells. Especially, the construction of CD19 antigen on the surface of aAPCs can selectively stimulate the expansion of CAR‐T cells, which expressed CD19 scFv.^17^, ^28^, ^29^, ^30^ The results showed that CAR‐T cells expanded in both ways had a similar morphology at the same time point, but the fold expansion of CAR‐T cells propagated with aAPCs was much greater than that by the beads. The CAR‐T cells stimulated by magnetic beads gradually disintegrated at around the 14th day, while the CAR‐T cells stimulated by aAPCs could survive longer and continue to be cultured to about 40 days (Data not shown). However, the best state of CAR‐T cells amplified with aAPCs could generally be observed after around 18 days, which might be as a result of the higher expression of exhausted molecules over time. Therefore, in the follow‐up experiments, the time points for comparing the two expansion methods were different. There was another study which reported that the shorter the culture time ex vivo, the better was the cell quality.^31^ Thus, further investigation was needed to determine the balance between the number of CAR‐T cells obtained and the state of the cells for best anti‐tumour effect.

The ratio of CD4^+^/CD8^+^ CAR‐T cells also affected the anti‐tumour response in vivo. When the ratio of CD4^+^/CD8^+^ CAR‐T cells was 1:1, it was the most homogeneous and effective cell product.^4^, ^23^, ^32^ In this study, the ratio of CD4^+^/ CD8^+^ CAR alive cells stimulated by aAPCs was 0.85, but that in cultures stimulated by beads was only 0.51. This meant that CD19‐specific CAR‐T cells co‐cultured with aAPCs seemed to be more suitable for adoptive immunotherapy in vivo. Some previous studies had proved that anti‐CD3/CD28 beads stimulation would preferentially expand CD4^+^ CAR‐T cells rather than CD8^+^ CAR‐T cells, but the specific mechanism was not fully clarified yet.^15^, ^33^ However, our results showed the opposite data that anti‐CD3/CD28 beads stimulated the growth of CD8^+^ CAR‐T cells more than CD4^+^ CAR‐T cells. Yuki *et al* ^16^ previously reported that short‐term stimulation of beads preferentially proliferated CD8^+^ T cells and increased the anti‐tumour response of CAR‐T cells. Thus, the different gene transfer methods and time of co‐culture would influence the ratio of CD4^+^/CD8^+^ CAR‐T cells. Although NK cells and NKT cells were detected in our cell products, the ratio of NK cells in cell population was below 5%, which would not affect the proliferation of CAR‐T cells.^34^ And NKT cells also had CAR expression that could show target killing effect on CD19‐positive tumour cells.

The proliferative capacity, endurance and memory function of CAR‐T cells were necessary for long‐term existence and anti‐tumour effect in vivo.^35^, ^36^ Numerous studies were devoted to improving the memory function and reducing exhaustion of adoptively transferred CAR‐T cells.^37^, ^38^, ^39^ CD45RO was one of the T_CM_ hallmarks. Under the two co‐culture conditions, the growth rate of CD45RO^+^ CAR‐T cells expanded by aAPCs was higher than those expanded by the beads, and the expression of exhaustion marker CTLA‐4 was lower, which meant that aAPC‐expanded CAR‐T cells might persist for longer time in vivo. There was another study comparing the abilities of K562 CD3/28/137L aAPCs and beads in expanding T cells or CAR‐T cells, and similar results were observed as our present study.^40^ However, our method had an advantage in modified aAPCs mimicked the natural antigen‐presenting cells such as dendric cells as a result of their expression of CD86, CD64, CD137L, etc Our aAPCs also expressed the antigen CD19, which could selectively and effectively stimulate growth of CD19^+^ CAR‐T cells. Furthermore, our study employed the PB transposon system to transfer the CAR gene, which was distinct from the other study using retroviral vector. The number of CD8^+^ CAR‐T cells in beads‐stimulated group was greater comparing with that in aAPCs‐stimulated group, which might lead to the significantly higher tumoricidal activities on the four tumour cell lines through in vitro cytotoxicity assay. Little tumoricidal effect was observed when wild‐type K562 cells were treated with CAR‐T cells, whereas a marked effect was shown when CD19 antigen was expressed in the former by transfection, indicating CAR‐T cells possessed targeting effects. For the first time, our study showed the similarities and differences on growth rate, CAR expression, sub‐population, memory phenotype and tumoricidal activities between anti‐CD19 CAR‐T cells expanded with CD19/CD64/CD86/CD137L/mIL‐15 aAPCs and those with anti‐CD3/CD28 mAb‐coated beads.

CAR‐T cells secreted large amounts of cytokines after incubation with tumour cells, showing that they were still functioning with little exhaustion, because exhausted T cells would reduce cytokine production.^41^, ^42^ The cytokines secreted by CAR‐T cells were mainly IFN‐γ, IL‐2 and IL‐10, which reflected there was greater sub‐population of CD4^+^ CAR‐T cells. The major severe side effect of CAR‐T therapy was cytokine release syndrome (CRS), which was closely related with IFN‐γ, IL‐6 and IL‐10.^43^ Patients with CRS were usually given tocilizumab to alleviate the side effect. However, the expression of IL‐6 and IL‐10 were not high in our present study. Therefore, it was worth noting that tocilizumab might be useless, while the large amount of IFN‐γ would rather require reasonable management.

IFN‐γ could induce STAT‐mediated apoptotic signalling pathway,^44^ and granzyme B and perforin could activate the caspase cascade to induce cell death.^45^, ^46^ Therefore, our results indicated that CAR‐T cells induced apoptosis in haematologic tumour cells through IFN‐γ/STAT1 pathway and granzyme B/ perforin/caspase cascades. Although CAR‐T cells showed significant anti‐tumour effects on NAMALWA xenograft mouse model, the tumour burden was not completely eliminated. The most probable cause was that the expression rate of the targeted antigen CD19 in NAMALWA cells was only 32.85% (Figure 5A); thus, some of the tumour cells could not initiate CAR‐T cells' response.

Taken together, the two methods to expand CAR‐T cells transfected by *piggyBac* transposon system had their own advantages and disadvantages. Our results showed a greater expansion of CAR‐T cells with aAPCs, together with a higher memory phenotype expression and lower exhaustion level comparing with the beads. Moreover, aAPCs‐CAR‐T cells showed significant anti‐tumour effects in vitro and in vivo through apoptotic‐related pathways. In view of the above, our anti‐CD19 CAR‐T cells could be considered a potential agent for clinical application in B‐cell lineage haematological malignancies.

## CONFLICT OF INTEREST

The authors declare that they have no competing interests with the contents of this article.

## AUTHOR CONTRIBUTION

**Li‐Rong Yang:** Data curation (equal); Methodology (equal); Software (equal); Writing‐original draft (equal). **Lin Li:** Conceptualization (equal); Methodology (equal); Project administration (equal); Supervision (equal); Writing‐original draft (equal); Writing‐review & editing (equal). **Ming‐Yao Meng:** Conceptualization (equal). **W. Wang:** Conceptualization (equal). **Song‐lin Yang:** Methodology (equal). **Yi‐Yi Zhao:** Methodology (equal). **Run‐Qing Wang:** Methodology (equal). **Hui Gao:** Methodology (equal). **Wei‐Wei Tang:** Methodology (equal). **Yang Yang:** Formal analysis (equal); Methodology (equal). **Li‐Li Yang:** Formal analysis (equal); Methodology (equal). **Li Wei Liao:** Conceptualization (equal); Funding acquisition (equal); Project administration (equal). **Zong‐Liu Hou:** Conceptualization (lead); Funding acquisition (lead); Project administration (lead); Supervision (lead).

## Supporting information

Supplementary Material

## Data Availability

The data that support the findings of this study are available.
