# Supplementary material for: Evaluation of piggyBac‐mediated anti‐CD19 CAR‐T cells after ex vivo expansion with aAPCs or magnetic beads
Source: J Cell Mol Med. 2020 Nov 22;25(2):686–700. doi: 10.1111/jcmm.16118 (PMC7812273; doi:10.1111/jcmm.16118)

Supplementary

Table S1. Sequence of specific PCR primers of different target genes

| *Genes* | Sequence(5′→3′) |
| --- | --- |
| LTR | Forward：CTGAGCCTGGGAGCTCTCTG |
|  | Reverse：TTCCACACTGACTAAAAGGGTCTG |
| *PD-1* | Forward：CATCGGAGAGCTTCGTGCTA |
|  | Reverse：TGACCACGCTCATGTGGAAG |
| *CTLA-4* | Forward：TGTGCGGCAACCTACATGAT |
|  | Reverse：TGGCCCTCAGTCCTTGGATA |
| *LAG-3* | Forward：CAGCTCAATGCCACTGTCAC |
|  | Reverse：GCTCCACACAAAGCGTTCTT |
| *GAPDH* | Forward：CTGGGCTACACTGAGCACC |
|  | Reverse：AAGTGGTCGTTGAGGGCAATG |

Figure S1. The ratio of NK and NKT cells in the whole cell population. (A) Expression of CAR on day1 after electroporation. (B&C) After stimulating CAR-T cells with aAPCs or beads, the ratio of NK and NKT cells in the whole cell population on the last day.

Fig. S1


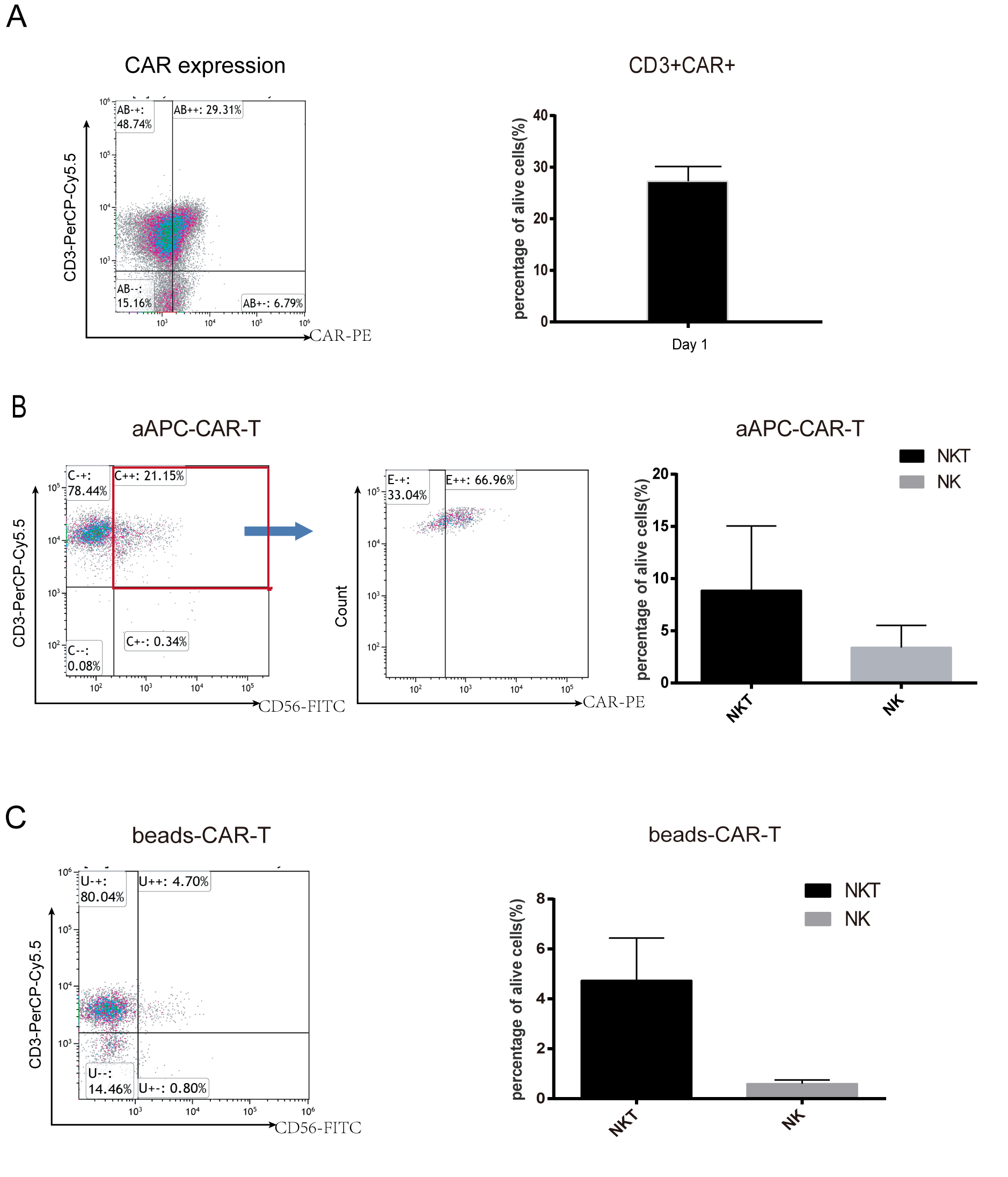

Supplement: Supplementary file 1 — Supplementary Material [file JCMM-25-686-s001.doc]
